# Protective Effect of Red Algae (*Rhodophyta*) Extracts on Essential Dietary Components of Heat-Treated Salmon

**DOI:** 10.3390/antiox10071108

**Published:** 2021-07-11

**Authors:** Jaime Ortiz-Viedma, José M. Aguilera, Marcos Flores, Roberto Lemus-Mondaca, María José Larrazabal, José M. Miranda, Santiago P. Aubourg

**Affiliations:** 1 Departamento de Ciencia de los Alimentos y Tecnología Química, Facultad de Ciencias Químicas y Farmacéuticas, Universidad de Chile, Santos Dumont 964, Santiago 8320000, Chile; rlemus@uchile.cl; 2 Departamento de Ingeniería Química y Bioprocesos, Facultad de Ingeniería, Pontificia Universidad Católica de Chile, Vicuña Mackenna 4860, Santiago 8940000, Chile; jmaguile@ing.puc.cl; 3 Departamento de Ciencias Básicas, Facultad de Ciencias, Universidad Santo Tomás, Talca 3460000, Chile; 4 Departamento de Ciencia de los Alimentos y Nutrición, Facultad de Ciencias de la Salud, Universidad de Antofagasta, Avenida Angamos 601, Antofagasta 1240000, Chile; maria.larrazabal@uantof.cl; 5 Departamento de Química Analítica, Nutrición y Bromatología, Facultad de Veterinaria, Universidad de Santiago de Compostela, 27002 Lugo, Spain; josemanuel.miranda@usc.es; 6 Department of Food Science and Technology, Marine Research Institute (CSIC), Calle Eduardo Cabello, 6, 36208 Vigo, Spain; saubourg@iim.csic.es

**Keywords:** red algae, antioxidant and antimicrobial ability, lipoperoxidation, salmon

## Abstract

Salmon paste contains nutritious components such as essential fatty acids (EPA, DHA), vitamin E and astaxanthin, which can be protected with the addition of red algae extracts. Phenolic extracts were prepared with an ethanol: water mixture (1:1) from the red seaweeds *Gracilaria chilensis*, *Gelidium chilense*, *Iridaea larga*, *Gigartina chamissoi*, *Gigartina skottsbergii* and *Gigartina radula*, obtained from the Pacific Ocean. Most algae had a high content of protein (>7.2%), fiber (>55%) and β-glucans (>4.9%), all expressed on a dry weight basis. Total polyphenols (TP), total flavonoids (TF), antioxidant (DPPH, FRAP) and antibacterial power of the extracts were measured. In addition, the nutritional components of the algae were determined. Results showed that the content of TP in the six algae varied between 2.6 and 11.3 mg EAG/g dw and between 2.2 and 9.6 for TF. Also, the extracts of *G. skottsbergii*, *G. chamissoi*, *G. radula* and *G. chilensis* showed the highest antiradical activity (DPPH, FRAP). All samples exhibited a low production of primary oxidation products, and protection of the essential components and the endogenous antioxidants tocopherols and astaxanthin, particularly in the case of *G. skottsbergii*, *G. chamissoi*, *G. radula* and *G. chilensis*. Furthermore, all algae had inhibitory activity against the tested microorganisms, coincident with their antioxidant capacity. Results show that the extracts may have future applications in the development and preservation of essential dietary components of healthy foods.

## 1. Introduction

For centuries, seaweeds have been an important source of entrenched food in Asian countries such as Japan, China, and Korea, and other coastal geographic areas in other regions of the world.

Although there is extensive harvesting of seaweeds at the artisanal level and the development of cultivation technologies has led to algae being a safe food, their use as staple foods and a source of bioactive components has not transcended to the western diet [1]. Many experts suggest that algae (seaweeds) can be an excellent alternative to the current trend of consumers to have natural and healthy plant-based foods and ingredients [1,2,3,4,5]. In recent times, there has been a wide interest in the functional properties of seaweeds and their application as bioactive components in the preparation of ingredients and additives that improve the conservation of different types of foods. Among the typical functional ingredients that are being investigated for their application in the formulation of healthy foods are antioxidants such as polyphenols, flavonoids, florotannins, carotenoids, etc., as well as immune protectants such as b-glucans, oligosaccharides, dietary fiber, lignans, peptides, etc. Moreover, seaweeds provide essential nutrients such as polyunsaturated fatty acids (PUFAS) and vitamins (folic acid and ascorbic acid, retinol), [2,3,6,7,8,9] and components related to sensory and gastronomic aspects that provide the umami flavor [1,9]. Therefore, the application of ingredients derived from algae may be a safe alternative to replace artificial food additives [10]. Recently, research regarding the use of red, green and brown algae extracts in the preservation of food has shown that the different bioactive antioxidant components derived from seaweed are effective in preserving fish and shellfish [11,12,13,14]. In addition, it has recently been observed that phenolic extracts from different red algae show the ability to inhibit the development of different pathogenic bacterial strains and the toxic products generated by their proteolytic activity, among which biogenic amines stand out due to their risk to human health [12,13,15]. Salmon is a fish of high nutritional richness due to its content in PUFAS, carotenoids, minerals, vitamins, etc., and is highly demanded by consumers. However, the high PUFAS content makes it very susceptible to deterioration by oxidation under refrigerated, frozen, cooked and dried conditions [16]. The presence of oxygen, metals, temperature, microbial contamination, manipulation, among others, are direct factors that catalyze the oxidative process of polyunsaturated lipids of marine species [14,16,17]. Therefore, it is necessary to search for natural antioxidants capable of inhibiting oxidation and the consequent loss of essential components (e.g., PUFAS, vitamins, amino acids, etc.) of salmon during thermal processing either as a processed product or as a culinary preparation.

The red seaweeds *Gracilaria chilensis*, *Gelidium chilense*, *Iridaea long*, *Gigartina chamissoi*, *Gigartina skottsbergii* and *Gigartina radula* occur in great abundance along the central and southern coast of Chile. They are normally exported at a low price to Asian countries without exploiting their use as food, functional ingredients and nutraceuticals at the local level. Given the many unknowns about the potential of chemical, bacteriostatic and food preservation properties of these algae species, the objective of this work was to develop a preliminary chemical study of the inhibitory power of phenolic extracts of these algae against lipid oxidation of salmon paste subjected to a cooking heat treatment. In addition, the ability of phenolic extracts to inhibit the development of different pathogenic bacterial strains was determined.

## 2. Materials and Methods

### 2.1. Red Algae Samples

**Figure 1:**
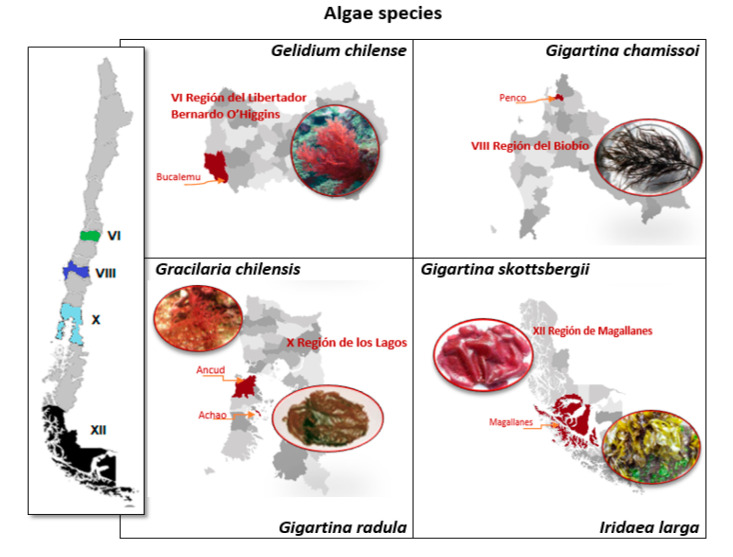
Red algae studied and region of Chile from which they were collected.

Six red algae species were collected from coastal areas in the central and southern regions of Chile (Figure 1) and dried at 60 °C for 3 h. Raw algae samples were provided by Algamar S.A. (Santiago, Chile). The dried algae were ground and sieved to a particle size of 0.8 ± 0.2 mm. Samples were stored at room temperature (25 °C) in sealed polyethylene bags.

### 2.2. Nutritional Content

The standard methods Association of Official Analytical Chemists (AOAC) and American Oil Chemists Society (AOCS) were used to quantify the moisture, ash, lipid and protein content in seaweed meals [18,19].. Considering that the sum of percentages of lipids, proteins, minerals and carbohydrates are equivalent to 100% of the dried algae. Once the percentages of lipids, proteins and minerals were determined, the carbohydrates were determined by subtraction (carbohydrates = 100 -% lipids -% minerals -% proteins). Total dietary fiber content was determined using half the amount of the algae sample and by applying AOAC procedures. The content of β-glucans was determined using an enzymatic assay (K-YBGL, Megazyme, Ireland) based on official methods [20]. Results were expressed as g/100 g dry basis.

### 2.3. Polyphenol Content

Hydroalcoholic extraction was used to obtain the phenolic extracts of algae. Dried algae (50 g) were soaked in 100 mL of extractant (ethanol/water: 50/50 *v*/*v*) and then stirred in a shaker (Wrist Action, Burell, Pittsburgh, PA, USA) for 8 h in a closed flask. Samples were then sonicated in a bath for 15 min at 25 °C, stirred for an additional 15 min, and filtered with a Whatman No. 1 paper. The entire procedure was repeated two more times but using 50 mL of extractant and only 30 min of shaking. The resulting extracts were finally concentrated at 40 °C in a vacuum rotary evaporator Büchi (Stuttgart, Germany) and then re-diluted with 200 mL of water (d) before storing at 5 ± 0.5 °C in glass bottles lined with aluminum foil. The total phenol (TP) of the algae was determined using the methodology proposed by Yildiz et al. (2011), which applies the Folin-Ciocalteu reagent and the colored solution was measured at 765 nm [21]. The TP of the extracts is expressed as micrograms of gallic acid equivalents per gram of dried algae (mg GAE/g dw).

### 2.4. Determination of Flavonoids

The flavonoids in the extract were measured by mixing 600 µL of the algae extract with 2.58 mL of solution A (1.8 mL of 5% NaNO2 and 24 mL of water). After standing for 5 min, 180 µL of 10% AlCl_3_ was added and the mixture allowed to stand for 1 min. Finally, 2.52 mL of solution B (12 mL of 1M NaOH plus 14.4 mL of water) was added and the contents were immediately read at 415 nm. Total flavonoids (TF) were obtained using a standard calibration curve of catechin (Sigma-Aldrich Co., Madrid, Spain) in a concentration range of 5 at 50 µg/mL and expressed as mg of catechin equivalents per gram of dried algae (mg CE /100 g dw) [22].

### 2.5. Antiradical Activity

Antiradical activity was evaluated by the DPPH test (2,2-diphenyl-1-picrylhydrazyl) radical assay according to Brand-Williams et al. (1995) [23]. Loss of color (purple) in the radical solution when exposed to each extract was measured at 517 nm. Results were expressed as % inhibition of DPPH radical according to the following formula:

DPPH (%) = [(Ac − As)/(Ac)] × 100(1)

where, Ac = control absorbance, As = Sample absorbance

### 2.6. Antioxidant Capacity by FRAP Method

The algae extracts were evaluated by the Iron Reduction/Antioxidant Power (FRAP) method [24]. The FRAP reagent was mixed in acetate buffer with the TPTZ solution and FeCl_3_ × 6 H_2_O. The mixture (1500 µL) was reacted at 37 °C with the extracts of algae 50 µL and 150 µL of water (d) After 4 min, the absorbance was measured at 593 nm. To determine the reduced Fe concentration, a FeSO_4_ calibration curve was used. Results were expressed in Mmol Fe^2+^/100 g.

### 2.7. Antibacterial Activity

To determine the antibacterial activity plating tests were carried out in agar wells according to the method proposed by González del Val [25]. The following bacterial strains were used: *Bacillus cereus* (ATCC 6633), *Escherichia coli* (ATCC 25922), *Staphylococcus aureus* (ATCC 25923), *Pseudomonas aeruginosa* (ATCC 27853), *Proteus mirabilis*, and a clinical strain of *Salmonella enteritidis*. These strains were grown in Mueller-Hinton broth for 24 h under agitation at 35 °C ± 2 °C. Isolated colonies were obtained for each strain. Bacteria were then streaked onto Mueller-Hinton agar plates (in triplicate) and grown for 24 h at 35 °C ± 2 °C. An isolated colony was selected from each inoculated dish and seeded into a tube containing a nutrient broth; this was followed by incubation at 35 °C ± 2 °C. Turbidity in the tubes was then adjusted to 0.5 units (108 CFU/mL) according to the McFarland standard. A sterile sachet (6 mm diameter) was used to make wells on the surface of agar plates. Prior to deposition into the wells, bacterial smears were prepared on Mueller-Hinton agar plates with the grass-planting technique and strain-dependent adjustments of bacterial concentration. Then, extract samples (25 μL) were deposited on each well (in triplicate), allowed to absorb for 30 min, and incubated for 24 h at 37 °C. Control tests were performed using pure (98% *v*/*v*) and diluted (50% *v*/*v*) methanol. The results were measured qualitatively by either the presence or absence of an inhibition halo.

### 2.8. Antioxidant Effect of Extracts in Cooked Salmon Paste

Cooked salmon pastes were prepared as follows. Salmon fillets obtained from the company Fiordo Austral (Puerto Montt, Chile) were kept in refrigeration after eliminating the inedible parts. Then the fillets were ground (Moulinex Mincer, AD6011, Shanghai, China) to obtain a homogeneous paste and kept refrigerated at 5 °C. Subsequently, extracts of each alga were prepared, dissolving 40 mg of dry extract in 50 mL of water (d). Each solution (50 mL) of extract was added individually in different samples of salmon paste (200 g each) to obtain a final concentration of 160 ppm of extract in the paste. This procedure was performed in triplicate. A paste control was prepared to which 50 mL of distilled water were added. A paste control was prepared to which 50 mL of distilled water was added. The pastes (10 g) with the addition of the algae extracts were placed in glass tubes and cooked for 30 min at 90 ± 5 °C by immersing them in a hot water bath. Subsequently, to determine the degree of oxidation of the lipid and loss of essential components (EPA, DHA, alpha-tocopherol and astaxanthin) from the heat-treated salmon muscle. Lipids were extracted from the muscle by the method of Bligh and Dyer [26].

### 2.9. Analysis of Polyunsaturated Fatty Acids ω-3 

To determine the protective effect of algae on polyunsaturated fatty acids w-3; EPA and DHA (eicosapentaenoic acid and docosahexaenoic acid) in the cooked salmon pasta, the identification and quantification of the fatty acids was carried out by gas chromatography (GLC), after extraction of the lipids from the fish paste by the method of Bligh and Dyer [26] and their derivatization to methyl esters.

The derivatization consisted of an alkaline methylation of 100 mg of oil added to 10 mL of 0.2 N sodium methylate under reflux conditions for 10 min. Followed by acid methylation (H_2_SO_4_/methanol) in boiling condition for 20 min, cooling and final extraction of the methyl esters with hexane followed by dissolution with 10% NaCL (Spanish Standard UNE 55-037-73). 

A gas chromatograph HP 5890 (Hewlett-Packard, Palo Alto, CA, USA) and a fused silica capillary column BPX70 (50 m, 0.25 µm film; SGE, Incorporated, Austin, TX, USA) were used. Conditions were: FID detector, injector and detector temperature 240 °C, initial temperature 160 °C, gradient 2 °C/min up to 230 °C. Carrier gas H2. FAME were identified based on sample standards (QualmixFish, Larodan, Malmo, Sweden; FAME Mix, Supelco, Inc., Merck, Darmstadt, Germany). In addition to determining the percentage content of the essential fatty acids EPA and DHA of the salmon paste, the polyunsaturation of the fat was expressed as a polyene index (PI) according to: PI = EPA + DHA/C16: 0 (palmitic acid).

### 2.10. Tocopherols

Measurement of tocopherols and provitamin E (α-Tocopherol) in the fat of salmon paste was carried out by HPLC analysis, with a fluorescence detector according to AOCS Official Method Ce 8-89 (1993) [19]. Detection was carried out at an excitation wavelength of 290 nm and emission 330 nm. The mobile phase was 2-propanol (0.05%) in hexane, with a flow of 1 mL/min. External standards α, β, γ and δ tocopherol (Merck, Darmstadt, Germany) were used and results expressed as µg tocol/g lipid.

### 2.11. Lipid Oxidation

The peroxide index (PV) was measured according to the AOCS Official Method, Cd 8-53 (1993) [19]. Five grams of extracted salmon fat were introduced into a 250 mL flask and mixed with 30 mL of acetic acid-chloroform solution (3:2), stirring the mixture with 3 g of KI and 0.5 mL of distilled water. The stoppered flask was shaken in the dark for 1 minute stopping the reaction with 30 mL of distilled water. The mixture was titrated with 0.01 N sodium thiosulfate and starch as indicator. The results were expressed in meq of active oxygen/kg of fat.

### 2.12. Statistical Analysis

Data from the different chemical assays were statistically analyzed using Statgraphics Centurion XV (StatPoint Technologies Inc., Warrenton, VA). Results were evaluated using analysis of variance (ANOVA). Mean values (n = 3) were compared through the multiple range test, using the procedure of honestly significant difference (HSD) of Tukey. In all cases, differences were considered significant at a confidence level of 95% (*p* < 0.05).

## 3. Results and Discussion

### 3.1. Nutritional Composition

Table 1 presents the nutritional composition of each algae species. Most algae presented low amounts of total lipids (0.2–1.4%), similar to percentages reported in some terrestrial edible plants, such as spinach (0.8%) and chard (0.4%) [27]. The obtained values also coincide with low lipid contents found in other macroalgae, where the reported maximum value is 4% of dry weight (dw) [28,29,30].

Algae presented notable differences in lipid contents, with the lowest and highest contents found in *G*. *skottsbergii* (0.20%) and *G*. *chamissoi* (3.73%), respectively. Variability in lipid contents may be due to differences between species and phenomena associated with growth conditions in each geographic zone. Likewise, protein contents varied widely. The highest protein contents were found in *G. chilense* (20.26%), and the lowest protein contents were found in *G*. *skottsbergii*. Nevertheless, all of the studied algae presented values that are comparable to some common foods in the human diet, such as eggs (13%) [27]. Previous reports have estimated up to 34% (dry weight) in protein contents of some seaweeds [30,31], which would be comparable to foods high in proteins, such as soy (36%). Protein contents are, however, dependent on species, collection time, and season, among other factors [28,32]. The percentage of total minerals in the six red algae varied between 8% and 25% with *G*. *skottsbergii* having the highest content and *G*. *chilense* the lowest. These results are lower than previous reports on mineral contents in algae. The mineral content in algae normally represents close to 35% dw [30,33,34], which is higher than found in most terrestrial plants (excepting spinach, which has a value similar to algae). Mineral content found in the studied algae suggest they would be an important source of microelements (e.g., Ca, Fe, Zn). Mineral accumulation is due to the ability of algae to selectively absorb inorganic substances from the ocean through surface polysaccharides [29,33].

The amount of total dietary fiber found in the analyzed samples was close to 60%, excepting *G*. *radula* (see Table 1). Fiber content in studied algae surpasses foods traditionally considered to be high in dietary fiber, such as chard (47.7%), spinach (47.3%), and raw carrots (48.74%) [32,33]. Results are in line with the range (25-75%) reported for different varieties of brown and red algae [2]. Similar values in red algae were reported by Jiménez-Escrig and Goñi (1999) [29], indicating that the primary contents of fiber would be soluble dietary fiber comprised of sulfated galactans, agar, or carrageenans. The amount of dietary fiber in the assessed algae surpasses daily recommended values for humans (i.e., 30 g/portion), serving as a determinant of the principal nutritional and physiological effects of consuming this marine plant. 

The six assessed algae were noteworthy concerning protein, mineral, and fiber contents. Lipid contents, in turn, were lower. This would translate into fewer calories, meaning that the assessed algae could be candidates in the development of new food products for the management of weight loss. All of the red algae studied had significant levels of β-glucans (Table 1), which represented 3-7% dw. β-glucans have been shown to have beneficial effects for the immune system, as they are essential for the intestinal microbiota, favoring human health [35,36]. Bobadilla et al. (2013) determined similar contents of β-glucans in Chilean algae, finding the highest levels of β-glucans in the fronds of brown algae [6]. It should be noted that the β-glucan values obtained for *G. chilensis* in the present study even exceeded the highest values reported by Bobadilla et al. (2013) [6]. Since it has been shown that the β-glucans of *G. chilensis* can activate the cellular immune system of lymphocytes, it has been proposed that this alga could reduce the mortality of fingerlings in aquaculture, particularly for species of high commercial value, such as salmon or trout [6,36].

### 3.2. Antioxidant Activity of Phenolic Extracts

Results obtained for total polyphenols expressed in gallic acid equivalents (Table 2) indicate that polyphenols content in the six assessed algae species vary between 2.6 and 11.3 mg GAE/100 g algae. The highest polyphenolic contents were found in *G. skottsbergii* and *G. chilense*. On the other hand, *I. larga* and *G. radula* presents an intermediate value, whereas the lowest contents were found in *G. chilensis* and *G. chamissoi*. The total polyphenol contents were lower than in other types of red algae, where values between 37 and 178 mg/g dw have been reported [15]. The flavonoid content for the six algae varied between 2.2 and 9.6. Both polyphenol and flavonoid contents were similar to those determined in the algae *Hypnea musciformis* and *Acanthophora muscoides* [15]. While the samples of *G. radula*, *G. skottsbergii* and *G. Chilense*, presented a greater antiradical capacity of DPPH according to their higher content of TP and TF (Table 2), they did not present a good reducing power of Fe^3+^. These results coincide with those obtained in other red algae studied by Arulkumar et al (2020) [15]. It should be noted that in most of the studies carried out on free radical or in lipid oxidation model systems, the antioxidant capacity is proportional to the polyphenol content [37,38,39,40]. Although *G. chamissoi* and *G. chilensis* did not have high levels of total polyphenols and flavonoids, they did present a good reducing value (FRAP).

Exceptionally, *I. larga* despite having a good level of polyphenols and flavonoids, did not present good anti-radical and reducing capacity. Some authors have reported that the higher the polarity of the solvents used, the higher the content of polyphenols extracted [40]. Thus, the polarity of the solvents used can help to produce a selective extraction of different bioactive compounds which have a different antiradical response capacity for the same type of sample. The previous treatment of the raw material could also cause the loss of the antioxidant power of the polyphenols present in these commercial red algae. In particular, the effect of drying could decrease the total content of polyphenols due to oxidation phenomena and structural changes, giving lower values than those present in fresh algae [29,37,40]. The most abundant polyphenols in algae known as florotannins, which can be in the form of floroglucinol polymers in various types of red, brown and green algae [37,41], could be affected by the conditions of extraction, drying, storage, among others. In addition, other bioactive components responsible for the antiradical properties such as catechins, flavonols and flavonol glucosides [42], which have been identified from methanolic extracts of red and brown algae could have been sensitive to the handling conditions of the samples. 

### 3.3. Antibacterial Assays

Table 3 presents the results obtained from the bacterial inhibition tests. The six hydroalcoholic algae extracts presented antimicrobial properties. A halo of inhibition was formed for most of the strains tested and over 90% of the plaques presented a halo of inhibition. The positive results of antimicrobial activity obtained for *G. radula*, *G. skottsbergii*, *G. chilense* and *G. chilensis* coincide with the high antioxidant capacities of these species. However, this result could be more related to the structure of the bioactive compounds than to the total content of polyphenols or antioxidant capacity. A similar inhibitory capacity has been observed in methanolic and acetone extracts from brown algae. More specifically, the methanolic extracts of *Sargassum latifolium* B and *Sargassum platycarpum* A were more active against Gram (+) and Gram (−) bacteria, the acetone extract of *S. latifolium* B being more inhibitory against *Salmonella* sp. [43]. Other studies with red algae extracts using methanol did not demonstrate bacterial inhibition but agglutinated Gram (+) bacteria from *S. aureus* and Gram (−) cells from E. *coli*, multi-resistant *Salmonella* and *Vibrio harveyi* [44].

The effectiveness of the red algae studied here may be due to the inhibitory action of other components present in the extracts such as pigments, organic acids and mineral salts, etc. Other studies in extracts of the red algae *Spergofusiforme* and *Sargassum vulgare* have used mass spectrometry identifying phenols, terpenoids, acetogenins, indoles, fatty acids and volatile halogenated hydrocarbons. Said extracts registered antimicrobial activity against the microorganisms *S. aureus* 2 and *Klebsiella pneumonia* [13] Among the studied algae, *I. larga* only presented antimicrobial activity against the stains *S. enteritidis*, *E. coli*, and *B. cereus* (Table 3). While this alga did not have the lowest total polyphenols contents, nor did it present the lowest antioxidant capacity, it is probable that the polyphenols present did not affect the bacterial strains due to the complexity of the bacterial cell membrane, which can be more selective and less permeable than the strains in which positive results were achieved.

The antimicrobial effect of algae extracts could be corroborated with transmission electron microscopy, looking at the morphological changes of microorganisms. For example, El Shafay et al. (2015) [13] observed that treatment of the bacteria *S. aureus* 2 and *K. pneumonia* with extracts from *S. fusiforme* and *S. vulgare* resulted in perforation of the cell wall, content-escape to the cytoplasm, and external distortion of the cell shape, among other damages to the cell structure [13]. Presumably, the bioactive components present in the six currently assessed red algae extracts would have permeated the interior of the microbial cell to produce cell-level damages, resulting in the inhibition of the bacterial strains used for assays.

### 3.4. Protective Effect of Lipid Oxidation in Cooked Salmon Paste

**Figure 2:**
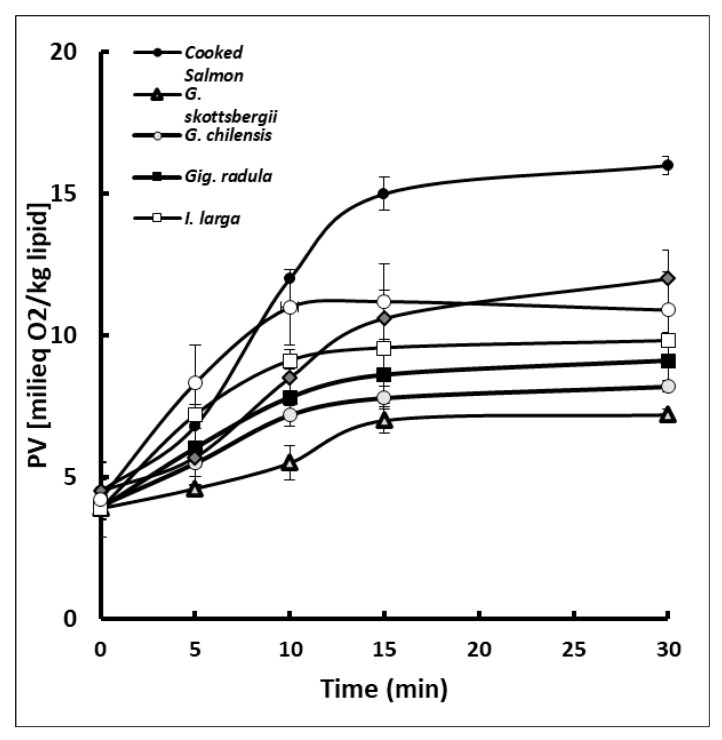
Inhibition of PV formation during cooking of salmon paste samples added with different extracts of red algae. Values are expressed as means (*n* = 3) ± standard deviation.

Figure 2 shows the inhibitory effect of red algae extracts on the oxidation of the fat of cooked salmon pasta, expressed by the peroxide number. Results indicated that all the algae extracts presented a protective effect against oxidation of salmon paste, given by an inhibition of the formation of peroxides that are primary oxidation products of lipids. The most efficient algae were *G. chamissoi*, *G. radula* and *G. chilensis*.

This protective effect was also observed in canned salmon prepared with liquid packaging containing extracts of the brown algae *Durvilleae antarctica*, *Ulva lactuca* and the red algae *Porphyra columbina* [14]. The protective effect can be attributed to the action of the bioactive components present in the seaweed extracts, such as, polyphenols, florotannins, diterpenes, carotenoids, phytosterols and possibly, low molecular weight hydrocolloids. On the other hand, extracts of other types of red and brown algae, added to chilled fish, have also shown effects against the oxidation and evolution of biogenic amines, trimethylamine, etc. [11,15,45,46].

Other plant-based foods that contain similar bioactive components have also shown protection in thermally processed fish [47,48]. The protective effect against thermal oxidation has been attributed to the solubilisation of phenols and other hydrophilic components at the water-muscle tissue interface, which could be maximized in a homogenized system such as the salmon paste used in this study.

### 3.5. Protective Effect of EPA and DHA (PUFAS) in Cooked Salmon Paste

**Figure 3:**
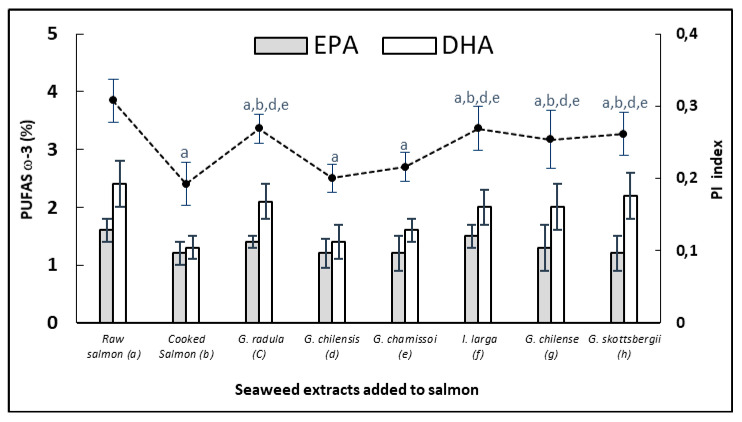
Polyene index of cooked and raw salmon pastes added with different extracts of red algae: (a) Raw salmon paste, (b) Cooked salmon paste, (c) *G. chamissoi*, (d) *G. skottsbergii*, (e) *G. radula*, (f) *I. larga*, (g) *G. chilense*, (h) *G. chilensis*. Values are expressed as means (*n* = 3) ± standard deviation. Different letters correspond to mean values with significant difference (*p* < 0.05).

Figure 3 shows the variation of the essential fatty acids EPA and DHA and the polyene index of the cooked salmon pasta samples versus the same pasta added with each of the red algae extracts. The results indicated that cooking the salmon paste without extracts generated a high decrease of DHA close to 50% and of 25% of EPA. 

On the other hand, most of the fish pastes treated with seaweed extracts presented a significant protection (*p* < 0.05) given by a high content of both essential fatty acids in the cooked product. Losses were reduced to less than 25 %, probably due to the inhibition of the fatty acid oxidation process in triglycerides, which was reflected in the maintenance of the polyene index. On the other hand, the percentage contents of EPA and DHA in the fat of salmon paste were as follows: (a) 1.6 ± 0.4; 2.4 ± 1.3; (b) 1.2 ± 0.2, 1.3 ± 0.1; (c) 1.4 ± 0.2, 2.1 ± 0.4; (d) 1.2 ± 0.3, 1.4 ± 0.3; (e) 1.2 ± 0.3, 1.6 ± 0.5; (f) 1.5 ± 0.3, 2.1 ± 0.1; (g) 1.3 ± 0.4, 2.0 ± 0.5; (h) 1.2 ± 0.3, 2.2 ± 0.6, respectively. C16: 0 remained constant at an average 10 ± 0.6. Similar results using plant extracts, algae and oils have been evidenced in other fish conservation studies at low and high temperatures [14,47,48].

### 3.6. Protective Effect of Pro Vitamin E in Cooked Salmon Paste

**Figure 4:**
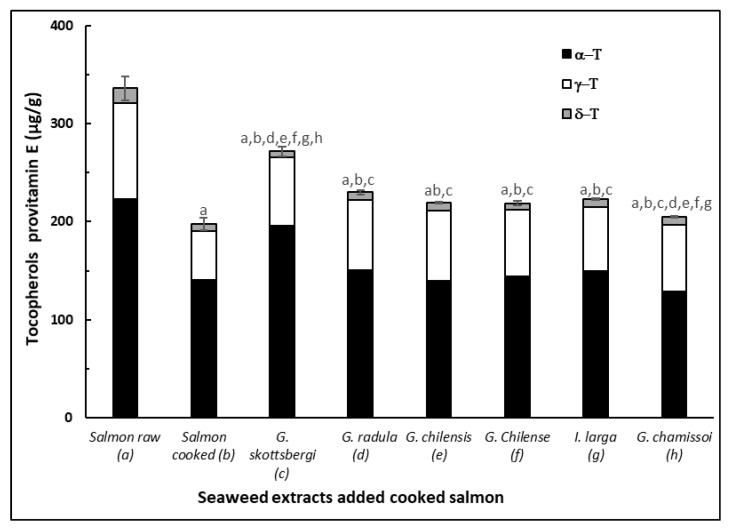
Tocopherols of cooked and raw salmon pastes added with different extracts of red algae: (a) Raw salmon paste, (b) Cooked salmon paste, (c) *G. chamissoi*, (d) *G. skottsbergii*, (e) *G. radula*, (f) *I. larga*, (g) *G. chilense*, (h) *G. chilensis*. Values are expressed as means (*n* = 3) ± standard deviation. Different letters correspond to mean values with significant difference (*p* < 0.05).

Figure 4 shows the level of tocopherols in control raw and cooked salmon pasta versus pastas added with red algae extracts and cooked at 90 °C for 30 min. Results indicated that in general the content of α, γ, δ-tocopherols had a significant loss (*p* < 0.05) due to cooking, presented in salmon paste cooked with seaweed extracts. On the other hand, all samples of salmon paste added with extracts of red algae significantly decreased the loss of tocopherols from the paste (*p* < 0.05) compared to the cooked control pasta. The algae *G. skottsbergii* maintained a higher content of tocopherols in salmon than the other red algae. The *G. chilensis* extract was the one that maintained a higher content of α, γ and δ-tocopherol. The other salmon pastes presented a similar content of tocopherols. Similar results have been observed in extracts of other types of vegetables such as stevia and grape skins applied to fish, shellfish and crustaceans [14,15,47,48,49,50].

Algae extracts may have contributed with tocopherols [29] in addition to flavonoids and polyphenols from the salmon pastes. Losses of tocopherols during cooking are due to their protective action against the thermo-oxidation of polyunsaturated lipids of the adipose tissue of salmon, which can be associated with the maintenance presented in the content of EPA and DHA and in the polyene index (PI) and (Figure 3). At the same time, these results are correlated with the inhibition of the primary products given by the attenuation of the peroxide curves (Figure 2). Similar results have been also obtained in canned salmon heat treated in the presence of red and brown algae [14].

### 3.7. Protective Effect of Astaxanthin in Cooked Salmon Paste

**Figure 5:**
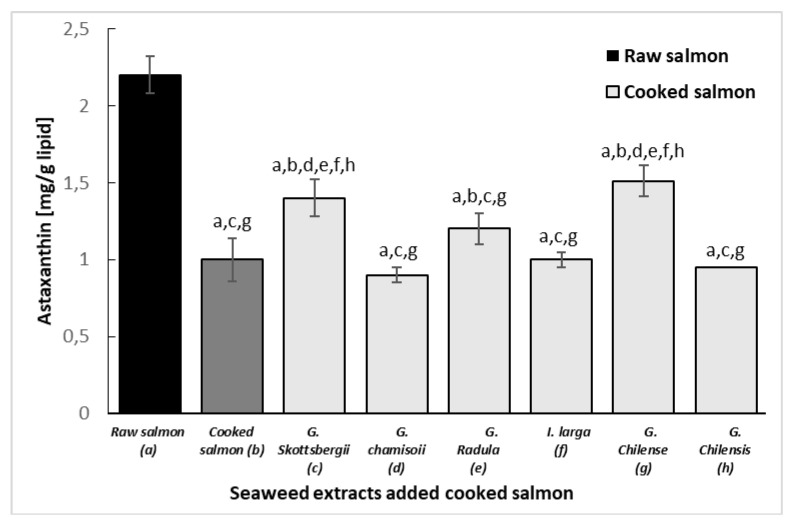
Astaxanthin of cooked and raw salmon pastes added with different extracts of red algae: (a) Raw salmon paste, (b) Cooked salmon paste, (c) *G. chamissoi*, (d) *G. skottsbergii*, (e) *G. radula*, (f) *I. larga*, (g), *G. chilense*, (h) *G. chilensis*. Values are expressed as means (*n* = 3) ± standard deviation. Different letters correspond to mean values with significant difference (*p* < 0.05).

Figure 5 shows the level of astaxanthin in raw and cooked salmon pasta versus the same pasta added with red algae extracts and cooked at 90 °C for 30 min. Results indicated a loss of more than 50% of astaxanthin in the control salmon paste. A moderate inhibitory effect of astaxanthin degradation was observed only in the samples with *G. skottsbergii*, *G. radula* and *G. chilense*. The relative protection of seaweed extracts against astaxanthin and tocopherols in cooked salmon pastes could be due to a synergistic effect of seaweed phenols and other endogenous antioxidant compounds from salmon muscle (such as ascorbic acid, uric acid, thiols, biliru-bin, etc.) capable of reversing the degradation of astaxanthin during lipid oxidation and the transition from tocopherol to tocopherylquinone. Therefore, part of the astaxanthin and tocopherol would be recovered and remain active, thus more effectively inhibiting the formation of peroxides and at the same time protecting the content of polyunsaturated fatty acids. [48,50,51].

## 4. Conclusions

This study suggests that beneficial dietary components present in red algae have some bioactive properties, especially a protective activity against the loss of essential dietary components present in salmon paste under thermo-oxidation conditions. The antioxidant activity and the protection of the essential fatty acids EPA and DHA, tocopherols (vitamin E) and astaxanthin present in salmon pastes, attributable to polyphenols, flavonoids and other bioactive components of the red algae, opens new possibilities of applications these extracts in fresh and processed foods. However, the evidence obtained requires of new studies to increase the available scientific knowledge on the functional and health-promoting properties of algae. In addition, it is necessary to deepen in sensory evaluation studies for marine products fortified with edible algae to determine the acceptability by consumers.

## Figures and Tables

**Table 1:** Nutritional composition of assessed algae species (g/100 g dw).

Algae	Ash	Lipids	Proteins	Carbohydrates	Fiber	β-Glucans
*I. larga (a)*	21.29 ± 1.69c	0.73 ± 0.19b	1.23 ± 1.45b	66.74 ± 4.49b	59.76 ± 2.09c	7.0 ± 0.4 b,d,f
*G. chilensis (b)*	12.28 ± 2.45b	1.36 ± 0.25c	19.94 ± 1.13d	66.42 ± 0.16b	59.99 ± 5.24c	4.9 ± 0.1 a,c,d,f
*G. chilense (c)*	8.77 ± 1.44a	1.40 ± 0.26c	20.26 ± 1.48d	69.57 ± 4.30a	55.45 ± 2.10b	6.0 ± 0.6 b,d,f
*G. chamissoi(d)*	13.66 ± 1.21d	3.73 ± 0.05d	14.08 ± 1.50c	68.53 ± 3.45a	55.16 ± 1.32b	3.2 ± 0.8 a,b,ce,f
*G. rádula (e)*	19.58 ± 1.52c	0.95 ± 0.14b	11.18 ± 1.18b	68.29 ± 2.20a	48.91 ± 1.71a	6.4 ± 0.2 b,d,f
*G.skottsbergii (f)*	25.72 ± 1.24b	0.20 ± 0.03a	7.57 ± 0.91a	66.51 ± 5.32d	59.18 ± 5.11c	5.6 ± 0.3 a,b,d,e

Values correspond to the average of triplicates ± standard deviation. Letters indicate significant differences (*p* < 0.05). Proteins were estimated by converting nitrogen content using a factor of 6.25. Total carbohydrates were estimated as the difference of subtracting 100 other components (i.e., lipids, minerals, proteins, moisture).

**Table 2:** Total polyphenols (TP), Total flavonoids (TF) contents and antiradical capacity (IC50) of algae extracts. Values correspond to the mean of three independent analyses ± standard deviation.

Algae	TP	TF	DPPH	FRAP
	mgGAE/g dw	mgCE/g dw	(%)	g Fe^2+^/100 g
*I. larga (a)*	6.9 ± 1.2 b,c,d,f	5.8 ± 1.2 b,c,d,f	26.6 ± 3.5 b,c,e,f	0.36 ± 0.01 b,c,d
*G. chilensis (b)*	2.6 ± 0.6 a,c,e,f	2.2 ± 1.2 a,c,e,f	34.2 ± 6.0 a,c,e,d,e	0.57 ± 0.11 a,c,d,e,f
*G. chilense (c)*	9.9 ± 1.3 a,b,c,d,e	8.4 ± 1.2 a,b,d,e,f	51.2 ± 9.1 a,b,c,d,e,d	0.47 ± 0.04 a,b,d,e,f
*G. chamissoi (d)*	3.4 ± 0.4 a,b,d,e,f	3.1 ± 1.2 a,b,c,d,e	21.6 ± 4.1 b,c,d,e	0.62 ± 0.08 a,c,d,f
*G. rádula (e)*	6.1 ± 0.9 a,b,c,d	5.1 ± 1.2 b,c,d,e	72.2 ± 10.8 a,b,c,d	0.31 ± 0.01 b,c,d
*G. skottsbergii (f)*	11.3 ± 2.1 a,b,c,d,e	9.6 ± 1.2 a,b,c,d,e	66.9 ± 16.4 a,b,c,d	0.34 ± 0.01 b,c,d

Values correspond to the average of triplicates ± standard deviation. Letters indicate significant differences (*p* < 0.05).

**Table 3:** Detected antimicrobial reactions of assessed algae extracts.

	Bacterial Strain					
Algae Extract	*S. enteritidis*	*E. coli*	*B. cereus*	*P. aeruginosa*	*St. aureus*	*P. mirabilis*
*Iridaea larga*	+	+	+	−	−	−
*Gracilaria chilensis*	+	+	+	+	+	+
*Gelidium chilense*	+	+	+	+	+	+
*Gigartina chamissoi*	+	+	+	+	+	+
*Gigartina radula*	+	+	+	+	+	+
*Gigartina skottsbergii*	+	+	+	+	+	+

+: Positive antibacterial activity. −: Negative antibacterial activity.

## Data Availability

Data is contained within the article.
